# Trunnionosis and prosthesis dissociation after total hip arthroplasty

**DOI:** 10.1016/j.radcr.2024.01.015

**Published:** 2024-01-20

**Authors:** Eva Napierkowski, Juhyun Lee, Nihal Thapa, Erin Brown, Yustina Salama, Emad Allam

**Affiliations:** a Loyola University Medical Center and Loyola University Chicago, 2160 S First Ave, Maywood, IL 60153 USA; b Tulane University School of Medicine, 1430 Tulane Ave, New Orleans, LA 70112 USA

**Keywords:** Trunnion failure, Trunnion dissociation, Total hip arthroplasty, Stryker Accolade TMZF, Trunnionosis, Metallosis

## Abstract

Femoral neck trunnion dissociations are rare complications of total hip replacements. This hardware failure is often due to underlying trunnionosis which is important to recognize. We present a case of a patient with dissociation at the femoral head-neck junction of a total hip arthroplasty (THA) with a Stryker Accolade TMZF femoral stem. There was no significant preceding trauma. The complication was visualized on radiography and confirmed during revision arthroplasty.

## Background

Total hip arthroplasty (THA) is an elective treatment option for debilitating hip deterioration caused by osteoarthritis, inflammatory arthritis, hip dysplasia, trauma, and more. With increasing levels of older patients with obesity and osteoarthritis in the United States, THA rates are expected to almost double from 2012 to 2030 [1]. Generally, over 90% of individuals who undergo THA are pain-free and without THA-related complications 15 years after surgery [2]. When postoperative THA complications do occur, they include loosening, particle disease, dislocation, periprosthetic fracture, and infection, among others.

Dissociation of the femoral head-neck junction is a rare complication of THA. Among the primary causes is metallic corrosion leading to trunnionosis, or wear of the femoral head-neck interface [3]. Mixed evidence indicates that larger diameter femoral heads, while decreasing the risk of dislocation, may increase the risk of head-neck wear [4,5,–6]. Mixed alloy couples may also be a risk factor for trunnionosis [7]. Patient weight also factors into the risk for trunnionosis, with increasing weight directly correlating with fretting wear [8].

Though dissociation of the femoral head-neck interface is uncommon, this type of hardware failure is important to identify so that safe, effective revision arthroplasty can be performed before further compilations arise.

## Case presentation

An 86-year-old male patient with a BMI of 32 kg/m² underwent a metal-on-polyethylene left total hip arthroplasty using a Stryker Accolade TMZF femoral stem in 2008. Fifteen years after the THA, when getting in or out of a vehicle with a high step, he heard a pop and noted the onset of left thigh and knee pain with a feeling of instability in his leg. He denied fevers, chills, or erythema, or drainage. He presented to clinic about a month later and examination revealed that when the patient stood, he could bear weight on the left leg, but it was considerably shorter than the right.

**Fig. 1:**
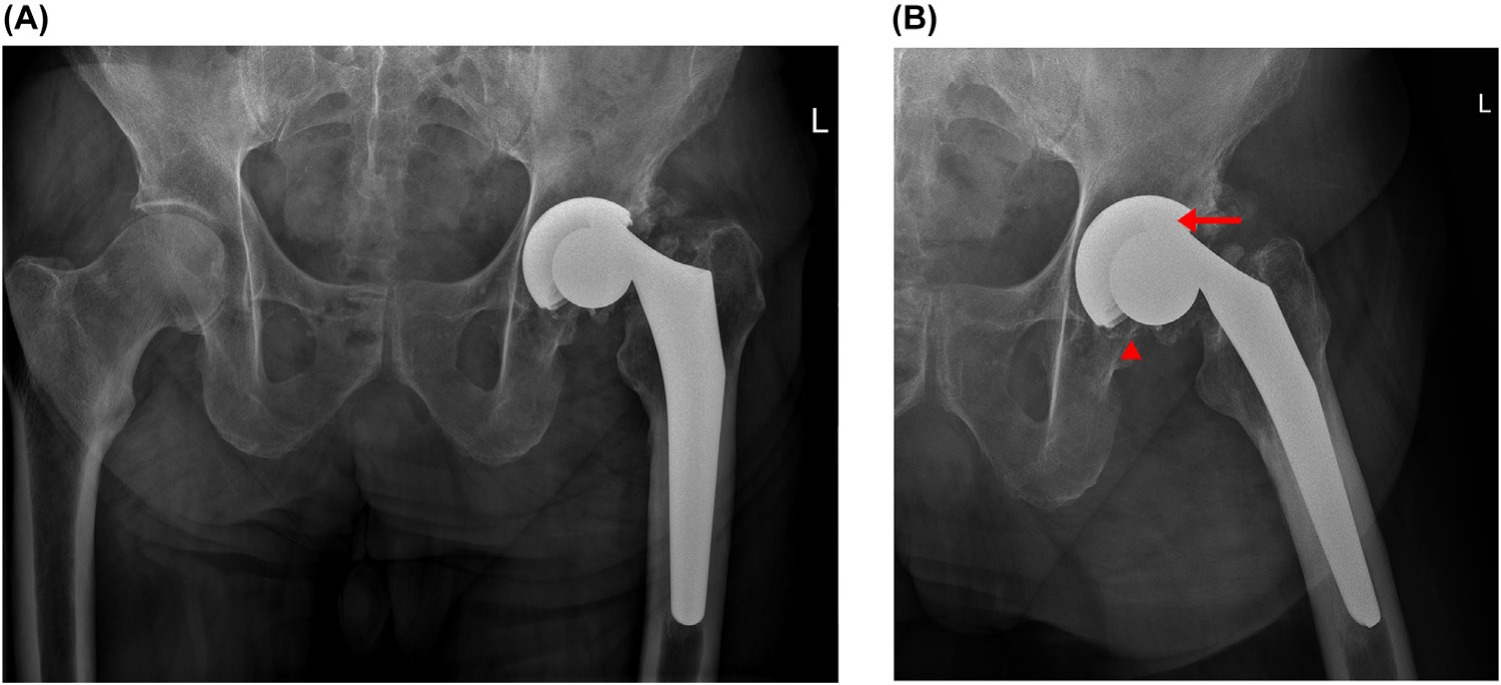
Radiographs of the (A) pelvis and (B) left hip demonstrate a metal-on-polyethylene design left total hip arthroplasty. There is dissociation of the prosthetic femoral head from the femoral stem. The trunnion is tapered and is superolateral relative to the femoral head, projecting into the region of the polyethylene liner (arrow). The femoral head component remains located within the acetabular component. Metallic debris is visible (arrowhead). Nonbridging heterotopic ossification is present at the superolateral aspect of the hip.

**Fig. 2:**
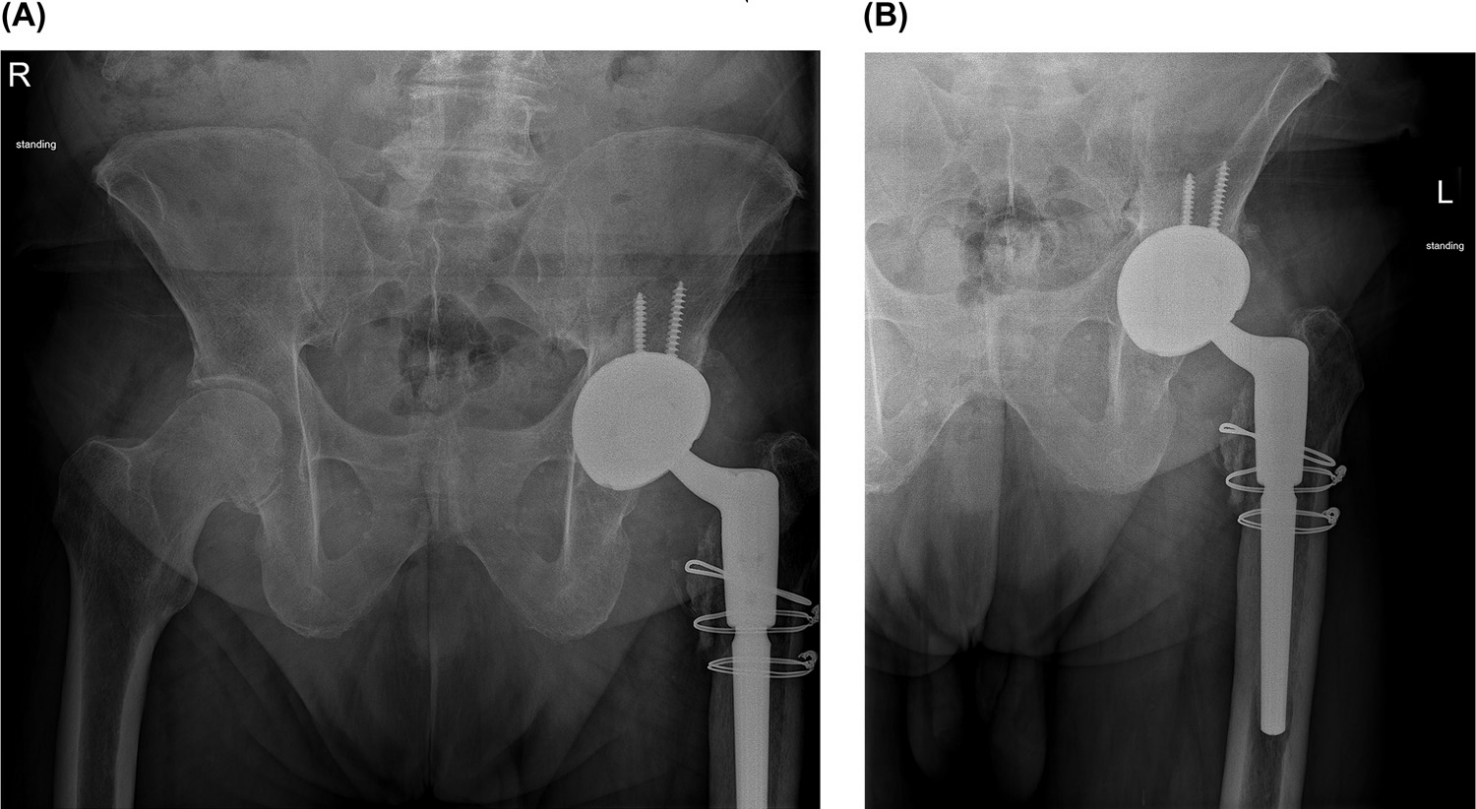
Radiographs of the (A) pelvis and (B) left hip obtained 6 months after revision surgery demonstrate revised left total hip arthroplasty with well-seated components. The left lesser trochanter fracture sustained during surgery and fixed with cerclage cables appears to be healing.

Radiographs at the time of the clinic visit demonstrated failure of the trunnion with dissociation of the femoral head from the trunnion (Fig. 1). The patient underwent revision surgery one week later. At the time of revision surgery, a large amount of metal debris was seen in the hip joint capsule. Other than scarring from the prior surgery, no surrounding soft tissue reactive changes were seen. The metal femoral head was dissociated from the trunnion. The trunnion appeared pencil-tip in nature consistent with catastrophic trunnion wear. Superior wear of the polyethylene liner was noted, which may have been due to articulation with the trunnion. Revision of both the acetabular and femoral components was performed. A fracture of the lesser trochanter occurred during stem removal, which was fixed with cerclage cables. The patient is doing well 6 months after revision surgery without evidence of hardware complications (Fig. 2).

## Discussion

Trunnionosis is defined as wear of the femoral head-neck interface; it is sometimes also referred to as mechanically assisted crevice corrosion (MACC). Studies reflect that up to 3% of THA revisions are due to trunnionosis [9]. The exact cause of trunnionosis is unclear, with possible etiologies including modular junction wear, corrosion damage, and metal ion release. Metallosis or adverse local tissue reaction (ALTR) or adverse reaction to metal debris (ARMD) is often thought of as a soft tissue reaction occurring due to metallic debris originating from the femoral head-acetabular articulation in metal-on-metal (MoM) hip arthroplasties. However, it can certainly also occur due to trunnionosis at the femoral head-neck junction of metal-on-polyethylene (MoP) hip arthroplasties.

Attempts to predict patients at most risk for trunnionosis after THA suggest that males with BMI > 30 kg/m^2^ are at greatest risk [9]. At a BMI of 32 kg/m^2^, our patient fits this profile. The Stryker Accolade TMZF stem has also been associated with this complication and has since been recalled.

In our patient, we postulate that trunnionosis resulted in significant wear of the implant which culminated in the dissociation of the implant, which the patient described as a popping sensation. Imaging demonstrated catastrophic trunnion failure, particularly dissociation of the prosthetic femoral head from the femoral stem. Differential diagnosis included fracture of the trunnion. However, it is important to recognize that even though discontinuity of the components on imaging is suggestive of fracture, this is rather a dissociation of the modular components. Wear and tapering of the trunnion eventually allow the prosthetic femoral head component to dissociate from the femoral neck with minimal trauma. ALTR may be seen in the surrounding soft tissues as a reaction to metallic debris in such cases.

## Conclusion

We present a case with classic patient demographic, history, and imaging findings indicative of trunnionosis with component dissociation. Trunnionosis is a known complication of THA that may result in prosthetic fracture, component dissociation, or ALTR, all of which are indications for revision surgery. It is important for radiologists to be aware of this complication and promptly recognize it on imaging.

## Patient consent

Informed consent for this case was obtained from the patient.
